# Beneficial Effects of Tea and the Green Tea Catechin Epigallocatechin-3-gallate on Obesity

**DOI:** 10.3390/molecules21101305

**Published:** 2016-09-29

**Authors:** Takuji Suzuki, Monira Pervin, Shingo Goto, Mamoru Isemura, Yoriyuki Nakamura

**Affiliations:** 1 Faculty of Education, Art and Science, Yamagata University, Yamagata 990-8560, Japan; taksuzuk@e.yamagata-u.ac.jp; 2 Tea Science Center, University of Shizuoka, Shizuoka 422-8526, Japan; monira689@yahoo.com (M.P.); yori.naka222@u-shizuoka-ken.ac.jp (Y.N.); 3 Graduate School of Nutritional and Environmental Sciences, University of Shizuoka, Shizuoka 422-8526, Japan; gotos@affrc.go.jp

**Keywords:** green tea, catechin, obesity, adipogenesis, lipogenesis, lipolysis, AMPK

## Abstract

Green tea has been shown to have beneficial effects against cancer, obesity, atherosclerosis, diabetes, bacterial and viral infections, and dental caries. The catechin (−)-epigallocatechin-3-gallate (EGCG) has shown the highest biological activity among green tea catechins (GTCs) in most of the studies. While several epidemiological studies have shown the beneficial effects of tea and GTCs on obesity, some studies have failed to do this. In addition, a large number of interventional clinical studies have shown these favorable effects, and cellular and animal experiments have supported those findings, and revealed the underlying anti-obesity mechanisms. One of the mechanisms is enhanced cellular production of reactive oxygen species, which is mediated through the pro-oxidant action of EGCG, leading to the activation of adenosine monophosphate-activated protein kinase, which suppresses gene and protein expression of enzymes and transcription factors involved in adipogenesis and lipogenesis, and stimulates those involved in lipolysis. Recently, scientific evidence supporting the beneficial anti-obesity effects of green tea and GTCs has been increasing. However, future investigations are still required to clarify the reasons for the inconsistent results reported in the human studies; to achieve this, careful adjustment of confounding factors will be required.

## 1. Introduction

Tea, a product obtained from the leaves and buds of the plant *Camellia sinensis*, is one of the world’s most popular beverages. Black, oolong, and green tea are all obtained from *C. sinensis* leaves through full fermentation, semifermentation, and non-fermentation, respectively [1,2,3]. Green tea was discovered in China in 3000 BC or earlier, and has been well recognized to have medicinal effects [1,2]. It was brought from China to Japan by Buddhist priests thousands of years ago. In 1211, a Japanese Zen priest, Yeisai, published the book “Kitcha-Yojoki” (Tea and Health Promotion), in which he described the methodology of harvesting tea leaves, the processes of tea production, and the pharmacological effects of tea. In the Edo period in Japan, medical doctor Ekiken Kaibara (1630–1714) stated in his book “Yojokun” (Lessons for Health Promotion) that long-term drinking of green tea is not beneficial as it removes body fat leading to weight loss [4]. However, this effect may be accepted nowadays because of its potential usefulness in the prevention of obesity.

**Figure 1:**
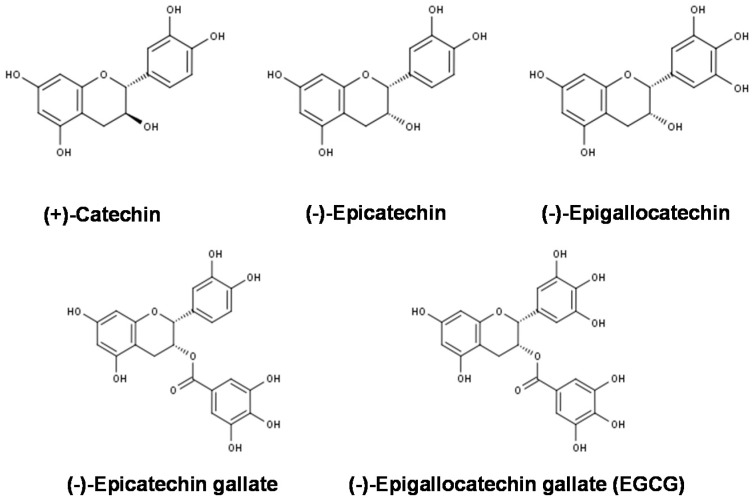
Chemical structures of (+)-catechin and major green tea catechins.

Several components of tea have specific health benefits [1,2,5,6,7,8]. Catechins (Figure 1), which are polyphenolic compounds, are associated with the anti-cancer, anti-obesity, anti-atherosclerotic, anti-diabetic, anti-bacterial, anti-viral, and anti-dental caries effects of tea [1,2,7,8]. Caffeine stimulates wakefulness, decreases the sensation of fatigue, and has a diuretic effect [2,7]. Theanine and γ-aminobutyric acid lower blood pressure and regulate brain and nerve functions [2,7]. Vitamin C is antiscorbutic, prevents cataracts, and strengthens the immune system [2,9]. This review will discuss briefly the anti-obesity effects of green tea and its catechins, especially (−)-epigallocatechin gallate (EGCG), which has the highest biological activity among green tea catechins (GTCs).

## 2. Epidemiological Studies

Several epidemiological studies have shown the beneficial effects of tea and its catechins on obesity [5,6,7,8]. For example, an early study in The Netherlands showed that high dietary intake of flavones, flavonols, and catechins was inversely associated with body mass index (BMI) in women [10].

An epidemiological study carried out between 2003 and 2006 on a sample of 6472 participants found that hot tea consumption was inversely associated with obesity; hot tea consumers had lower mean waist circumference and lower BMI than non-consumers did. In contrast, iced tea consumption was found to be associated with higher BMI, greater waist circumference, and greater subcutaneous skinfold thickness after adjustment of confounding factors, e.g., sugar intake [11].

A cross-sectional, population-based survey of 8821 adults conducted in Poland found that high tea consumers (3 cups/day) had lower BMI and waist circumference, but higher diastolic blood pressure, than low tea consumers did. The odds ratio for tea consumption with metabolic syndrome was 0.79, and among metabolic syndrome components, tea consumption was negatively associated with obesity and fasting plasma glucose in women, but not in men [12].

Gyntelberg et al. conducted a cross-sectional study on 3290 men aged 53–75 years, and found that 291 of them (8.8%) were obese and had a BMI ≥ 30. The prevalence of obesity was 6.2% among the men using sugar in coffee or tea, while it was 10.3% among others, i.e., an inverse association was observed between the use of sugar in hot beverages and the prevalence of obesity, and it was consistent in the subgroups [13]. This study indicates, rather surprisingly, that ingestion of small amounts of sucrose several times a day can have a weight-controlling, or reducing, effect.

In contrast, the results of a cross-sectional study that enrolled 554 adults in Tokushima, Japan, showed that green tea consumption was not associated with the prevalence of metabolic syndrome or any of its components [14].

Thus, epidemiological studies have provided conflicting results, which may have resulted from insufficient adjustment of confounding factors, such as the estimation method of tea consumption, cigarette smoking, and alcohol consumption [1,2,15]. Different results may have also arisen from differences in tea temperature, in view of the temperature-dependent results mentioned above [11]. In addition, caffeine consumption is an important factor to be adjusted, because GTCs and caffeine synergistically enhance sympathetic nervous system activity, leading to the increase in energy expenditure by fat oxidation [16]. In addition, intestinal microbiota and genetic polymorphisms may have influenced the effects of tea in these studies [7,8,17].

## 3. Clinical Trials

Many interventional clinical studies have been conducted to examine the effect of green tea and GTCs on obesity, and most of them have shown favorable effects [5,6,7,8]; however, some studies failed to demonstrate such effects, giving rise to the need for further studies.

In an early study on the anti-obesity effects of green tea, Tokimistu and his colleagues conducted a 12-week, double-blind human experiment, in which the subjects ingested one bottle of oolong tea containing either 690 mg of GTCs (GTC group) or 22 mg of GTCs (control group) per day. The results indicated that body weight, BMI, waist circumference, body fat mass, and subcutaneous fat area were significantly lower in the GTC group than those in the control group, suggesting the beneficial effect of GTCs on obesity [18]. A similar conclusion was drawn from a double-blind, randomized, controlled study conducted on obese or near-obese Japanese children, where the results indicated that consumption of a catechin-rich beverage ameliorated serious obesity and cardiovascular disease risk factors, without raising any safety concerns [19].

Another study showed that GTCs enhanced exercise-induced abdominal fat loss in overweight and obese adults. Participants received either a beverage containing approximately 625 mg of GTCs with 39 mg caffeine (GTC group) or a control beverage containing 39 mg caffeine with no GTCs (control group) for 12 weeks. The results showed greater body weight loss in the GTC group than in the control group. Although changes in fat mass did not differ between the two groups, percentage changes in total abdominal fat area, subcutaneous abdominal fat area, and fasting serum triglycerides were greater in the GTC group [20].

Similarly, the results from a different research group showed that green tea consumption, combined with resistance training, decreased body fat, waist circumference, and triglyceride levels, and increased lean body mass and muscle strength [21]. In a randomized clinical trial in which 18 patients with multiple sclerosis participated, supplementation of EGCG (600 mg/day) improved muscle metabolism during moderate exercise to a greater extent in men than that in women [22]. During exercise, postprandial energy expenditure was lower after EGCG than after placebo ingestion. The results of a randomized, double-blind, placebo-controlled, crossover pilot study showed that six overweight men administered 300 mg EGCG per day for 2 days had lower respiratory quotient values than those administered a placebo control, during the first postprandial monitoring phase [23]. This finding suggests that EGCG alone has the potential to increase fat oxidation and may contribute to the anti-obesity effects of green tea.

In a randomized, placebo-controlled trial, 182 moderately overweight Chinese subjects were divided into four groups: control, GT1, GT2, and GT3 groups. The control subjects consumed two servings of a control drink (30 mg GTCs, 10 mg caffeine/day); the GT1 subjects consumed one serving of the control drink and one serving of an extra high-catechin drink (458 mg GTCs, 104 mg caffeine/day); the GT2 subjects consumed two servings of a high-catechin drink (468 mg GTCs, 126 mg caffeine/day); and the GT3 subjects consumed two servings of the extra high-catechin drink (886 mg GTCs, 198 mg caffeine/day) for 90 days [24]. The results showed that the GT3 group demonstrated significant reductions in intra-abdominal fat, waist circumference, and body weight than the control group did. In addition, reductions in total body fat and body fat percentage were observed in the GT2 and GT1 groups, respectively.

In an 8-week clinical trial, 35 obese subjects with metabolic syndrome were randomly assigned to the control (4 cups water/day), green tea (4 cups/day), or green tea extract (GTE) (2 capsules and 4 cups water/day) groups. Body weight and BMI significantly decreased in subjects who consumed green tea and GTE capsules than in the control group subjects, suggesting the improving effect of GTE on metabolic syndrome in obese patients [25].

Purple tea is a variety of green tea developed in Kenya; the major constituents of its leaves are caffeine, theobromine, epigallocatechin, EGCG, and 1,2-di-*O*-galloyl-4,6-*O*-hexahydroxydiphenoyl-β-d-glucose. Shimoda et al. showed that purple tea improved obesity parameters, including body weight, BMI, and body fat mass, in humans who ingested a tea infusion extracted from 1.5 g tea leaves with 100–200 mL hot water, twice a day for 4 weeks [26].

In a randomized, placebo-controlled, double-blind, crossover study, participants received either a beverage containing 55 mg of black tea polyphenols or a control beverage with no black tea polyphenols, 3 times/day for 10 days. Results of the fecal lipid measurements showed that the total lipid excretion increased from 5.51 g to 6.87 g/3 days after the intake of black tea polyphenols when compared with intake of the control beverage. Thus, black tea polyphenols may be useful for the prevention of obesity [27].

In a randomized, double-blind trial, 115 women with central obesity were randomly assigned to either a high-dose GTE group or a placebo group. Treatment with high-dose GTE for 12 weeks resulted in significant weight loss, reduced waist circumference, and decreased plasma levels of total cholesterol and low-density lipoprotein [28].

In another clinical trial, patients with type 2 diabetes ingested green tea with either 582.8 mg of GTCs (GTC group) or 96.3 mg of GTCs (control group) per day for 12 weeks. The results showed that the decrease in waist circumference was significantly greater in the GTC group than that in the control group. Adiponectin, which is negatively correlated with visceral adiposity, increased in the GTC group. These findings suggest that a catechin-rich beverage might have several therapeutic uses for the prevention of obesity [29].

A recent systematic review and meta-analysis revealed that green tea supplementation has a favorable effect on blood pressure in overweight and obese adults. Results obtained from the pooled analysis of 14 randomized controlled trials, with 971 participants, showed that supplementation of green tea, or GTE, resulted in significant reduction in both the systolic (−1.42 mmHg) and diastolic (−1.25 mmHg) blood pressures, as compared with those in the placebo group [30]. Several studies support this finding, while some show conflicting results as reviewed by Li et al. [31].

In a pilot study examining how GTE-enriched rye bread can control body weight and affect abnormalities related to metabolic syndrome, 55 obese men and women were recruited. This single-blind, randomized study showed that the ingestion of 280 and 360 g of GTE-enriched bread provided daily totals of 123.2 and 158.4 mg of caffeine, and 188.3 and 242.1 mg of EGCG for women and men, respectively. The study showed that GTE-enriched bread did not significantly influence the maintenance of waist loss or the concentrations of high-density lipoprotein, triglycerides, or glucose. However, it caused significant reduction in waist circumference (−1.22 cm), and the maintenance of lower blood pressure in the intervention group, compared with the control group [32].

In a crossover interventional study, Brown et al. observed that supplementation of decaffeinated GTCs decreased body weight by 0.64 kg, while the placebo group showed an increase of 0.53 kg in body weight, suggesting a preventive effect of GTCs on weight gain. Thus, it can be concluded that GTCs are responsible for the anti-obesity effects of green tea, although caffeine may also contribute to this effect [33]. Dulloo et al. showed that GTE had thermogenic properties and promoted fat oxidation to a degree beyond that ascribable to caffeine alone, suggesting that GTE may also be useful for the control of body composition via sympathetic activation of thermogenesis or fat oxidation or both [34]. The results of a meta-analysis have confirmed that catechin-caffeine mixtures, or caffeine-only supplementation, stimulate daily energy expenditure, and it can be concluded that both GTCs and caffeine contribute to the anti-obesity effects of green tea [35].

In contrast, several studies failed to show favorable anti-obesity effects of GTCs. For example, in a randomized, double-blind, placebo-controlled study, 83 obese, premenopausal women consumed 300 mg of either EGCG or placebo per day. The results showed that dietary supplementation of EGCG for 12 weeks did not enhance energy-restricted, diet-induced adiposity reductions, and did not improve weight-loss-induced changes in cardiometabolic risk factors in obese women [36].

In the Minnesota 12-month randomized, double-blind, placebo-controlled clinical trial, 937 postmenopausal women received either decaffeinated GTE, containing 843 mg of EGCG, or placebo. A sub-study was also conducted on 121 or 237 overweight/obese participants with a BMI of ≥25.0. In the GTE group, tissue fat percentage decreased during the intervention, although baseline BMI increased. In conclusion, decaffeinated GTE was not associated with reductions in body weight, BMI, or adiposity in overweight/obese postmenopausal women, but it was beneficial for reduction in tissue and gynoid fat percentages [37,38].

## 4. Cell-Based and Animal Experiments

Several cell-based and animal experiments have shown beneficial effects of green tea and GTCs on obesity [4,5,6,7]. For example, some studies showed that EGCG could decrease cell viability, inhibit 3T3-L1 cell differentiation, and decrease lipid accumulation [39]. In male mice with high-fat diet-induced obesity, acute oral administration of EGCG had no effect on body temperature and energy expenditure, but respiratory quotient during the night decreased, suggesting decreased lipogenesis and increased fat oxidation [40]. Bose et al. showed that supplementation of EGCG (3.2 g/kg) in the diet of mice fed a high-fat diet for 16 weeks reduced body weight gain, body fat percentage, and visceral fat weight than in untreated mice [41]. The results also indicated that EGCG treatment attenuated the development of obesity, symptoms associated with metabolic syndrome, and fatty liver [41]. Heber et al. showed that polyphenols of green, black, and oolong tea reduced visceral fat and inflammation in murine models of obesity induced by high-fat, high-sucrose diets [42].

In another study, the anti-obesity effects of three major components of green tea (GTCs, caffeine, and theanine) were examined in female ICR mice fed diets containing 2% green tea and diets containing 0.3% GTCs, 0.05% caffeine, and 0.03% theanine, alone and in combination, for 16 weeks [43]. The results showed that body weight gain and intraperitoneal adipose tissue weights were reduced by the diets containing GTCs, caffeine, theanine, caffeine + GTCs, caffeine + theanine, and caffeine + GTCs + theanine. The hepatic triglyceride level was significantly reduced by GTCs and GTCs + theanine, than by the control. These results suggest that caffeine and theanine are also responsible for the suppressive effect of green tea powder on body weight gain and fat accumulation. In addition, a combination of GTCs and caffeine demonstrated synergistic anti-obesity activities, and another experiment also demonstrated the possible anti-obesity effect of theanine [43]. In conclusion, most of the cell-based and animal experiments demonstrated beneficial effects of tea, and its constituents, on obesity. Some experiments also suggested that this effect might be enhanced by combinations of tea constituents, giving rise to a need for further studies.

## 5. Molecular Mechanisms

As discussed comprehensively by Yang et al., there are at least two major mechanisms involved in the action of green tea and GTCs, classified into direct and indirect mechanisms [8]. The direct mechanism includes effects on the digestive organs, such as prevention of absorption, inhibition of digestive enzymes, and changes in microbiota, while the indirect mechanism is mediated through modulation of gene expression, protein expression, and signal transduction in various tissues, including the liver, muscle, and adipose tissues.

### 5.1. Inhibition of Digestive Enzymes and Prevention of Absorption

Several studies have shown that GTCs could prevent absorption and inhibit digestive enzymes, eventually attenuating obesity. For example, Unno et al. found that in rats fed dietary GTCs (1% *w*/*w*), body and abdominal adipose tissue weights decreased after 4-week feeding periods, compared to those in the control rats [44]. Only 0.1% of the ingested starch was excreted in the feces of the control rats, whereas 4.8% was excreted in the feces of the GTC group. Apparent digestibility values of both lipids and proteins in the GTC group were lower than those in the control group, suggesting that GTCs increased the fecal excretion of these energy nutrients. Consistent with this finding, EGCG and epicatechin gallate were demonstrated to inhibit pancreatic lipase [45,46].

Ikeda et al. found that lymphatic recovery of ^14^C-trioleoylglycerol in rats with thoracic duct cannulation was delayed by the administration of GTCs. Experiments using individual components of GTCs showed that only galloylated catechins suppressed postprandial hypertriacylglycerolemia, by slowing down triglyceride absorption through the inhibition of pancreatic lipase [47].

Fei et al. found that EGCG, 3′′-*O*-methylated EGCG, and oolong tea polyphenols exhibited inhibitory effects against pancreatic α-amylase, and that their half-maximal inhibitory concentration (IC_50_) values were 0.350, 0.572, and 0.375 mg/mL, respectively [48]. Hot compressed water extract of black tea contains lipase-inhibiting polyphenols, and may be used as the source for potential anti-obesity dietary supplements and medications [46].

When a ^13^C-labeled mixed triglyceride breath test was performed, with and without GTE ingestion, in 32 healthy volunteers aged 23–30 years with normal exocrine pancreatic function, the cumulative percentage dose recovery value was 36.8% in the placebo group. This value was significantly higher than that of the GTE group (28.8%), indicating that GTE decreases lipid digestion and absorption in humans [49].

In male C57BL/6J mice fed a high-fat diet, green and black tea supplementation suppressed body weight gain and deposition of white adipose tissue, by stimulating glucose uptake and upregulating the expression of glucose transporter-4 on the plasma membrane of muscle cells [50]. Thus, as discussed by Yang et al., the anti-obesity actions of green tea in high-fat diet-induced obesity include suppression of absorption, upregulation of glucose transporter-4, and inhibition of digestive enzymes [8].

### 5.2. Effects on Intestinal Microbiota

In a study, rats were divided into three groups and fed either a control diet, a 0.3% EGCG diet, or a 0.6% EGCG diet for 4 weeks. The results showed that the 0.6% EGCG group showed a significant increase in fecal starch and protein contents, while the relative weights of abdominal adipose tissues were suppressed, than those in the control group. EGCG reduced the population of *Clostridium* spp. and increased that of *Bacteroides*, and, to a lesser extent, influenced the status of *Bifidobacterium* and *Prevotella*. Thus, dietary EGCG affects the growth of certain species of gut microbiota, which may be responsible for regulating energy metabolism in the body [51]. This and other effects of EGCG on intestinal microbiota have been reviewed by Yang et al. [8].

### 5.3. Effects on Gene and Protein Expression

Numerous cellular and animal studies have shown the effects of green tea and GTCs on the expression of obesity-related genes and proteins [6,7,8]. In a pioneering investigation, Murase et al. found that a catechin-rich diet enhanced hepatic gene expression of acyl-CoA oxidase (ACO) and medium-chain acyl-CoA dehydrogenase (MCAD), thus increasing hepatic β-oxidation activity and reducing fat accumulation [52].

**Figure 2:**
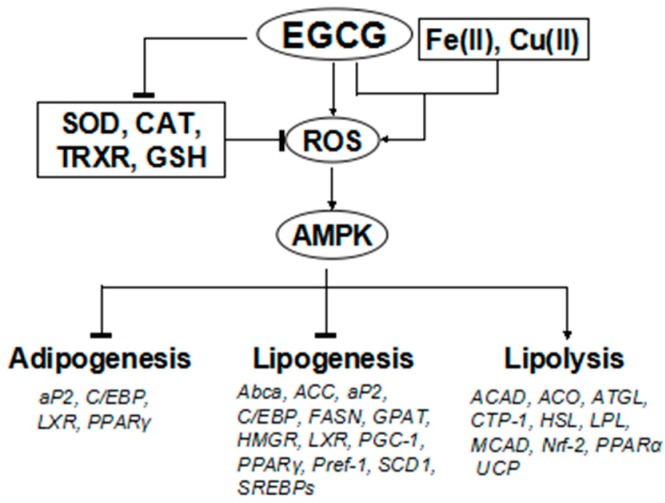
Effects of (−)-epigallocatechin-3-gallate (EGCG) on lipid metabolism via reactive oxygen species (ROS) and AMP-regulated protein kinase (AMPK). EGCG acts as a pro-oxidant, enhancing the generation of ROS, which activate AMPK [7,8,58,59,60]. AMPK activation suppresses adipogenesis and lipogenesis, while it increases lipolysis by regulating gene and protein expression of various enzymes and transcription factors, leading to the anti-obesity effect. Transition metal ions such as Fe(II) and Cu(II) may contribute to EGCG’s ROS-generating activity [61,62]. EGCG may also increase cellular ROS levels by decreasing the levels of antioxidant molecules, such as glutathione (GSH), and antioxidant enzymes, such as superoxide dismutase (SOD), catalase, and thioredoxin reductase (TRXR) [63,64,65]. Abca, ATP-binding cassette superfamily of transporter proteins; ACAD, acyl-CoA dehydrogenase; ACC, acetyl-CoA carboxylase; aP2, adipocyte protein 2; ATGL, adipose triglyceride lipase; GPAT, glycerol phosphate acyltransferase; LPL, lipoprotein lipase; Nrf-2, nuclear factor erythroid-2-related factor-2; PGC-1, peroxisome proliferator-activated receptor gamma coactivator-1; Pref-1, preadipocyte factor-1; SCD1, stearoyl-CoA desaturase-1; UCP, uncoupling protein.

Other studies have shown that green tea and GTCs suppress the expression of genes and proteins involved in adipogenesis, including CCAAT/enhancer-binding protein alpha (C/EBPα), peroxisome proliferator-activated receptor gamma (PPAR-γ), and liver X receptor alpha (LXR-α), and lipogenesis, including fatty acid synthase (FASN), hydroxymethylglutaryl-CoA reductase (HMGR), acetyl-CoA carboxylase (ACC), sterol regulatory element binding protein (SREBP)-1c, and stearoyl-CoA desaturase-1 (SCD-1) (Figure 2). However, they stimulate those involved in fatty acid mobilization, such as ACO, hormone sensitive lipase (HSL), adipose triglyceride lipase (ATGL), and PPAR-α (Figure 2) [5,6,7,8]. Yang et al. proposed “the AMPK hypothesis” to highlight the central role of AMP-regulated protein kinase (AMPK), which leads to either downregulation or upregulation of expression of various genes (Figure 2) [8].

Among these proteins, SREBPs have a key role in the regulation of the expression of genes related to lipid biosynthesis, such as *ACC*, *FASN*, *HMGR*, and *SCD-1* [15,53]. Therefore, it is possible that GTC’s suppressing effects on these genes occur through suppression of the expression of SREBPs.

Consistent with the “AMPK hypothesis”, Murase et al. postulated that, based on in vivo and in vitro experimental results, many of the GTCs’ effects, including anti-obesity and anti-cancer effects, are mediated by the activation of the liver kinase B1/AMPK in various tissues [54]. In an experiment using an obese mouse model fed a high-fat diet, decaffeinated GTE attenuated diet induced-increases in body weight gain, and increased serum adiponectin levels. The GTE group showed an increase in hepatic protein expression of phosphorylated AMPK, which can explain the observed decrease in hepatic protein expression of ACC, FAS, SREBP-1, and carbohydrate response element-binding protein [55].

Treatment of pre-adipocyte 3T3-L1 cells with EGCG resulted in decreased expression of the adipocyte marker proteins PPAR-γ2 and LXR-α, and induced generation of reactive oxygen species (ROS), which activate AMPK [39]. As described above, consumption of purple tea improved obesity parameters in humans, and similar effects were observed in an animal experiment. Protein expression of carnitine palmitoyltransferase (CTP)-1A was enhanced in the liver of mice administered purple tea. This enhancement was also observed in cultured HepG2 hepatoma cells treated with purple tea. In conclusion, purple tea may exert an anti-obesity effect through enhancement of fatty acid oxidation [26].

### 5.4. Effects on the Expression of Selected Genes and Action Mechanism of Green Tea Constituents

We previously showed that chronic administration of catechin-rich green tea reduced hepatic gene expression of FASN in normal rats [56], which can be explained by the “AMPK hypothesis.” In a later study, we observed that administration of an ethyl acetate-insoluble fraction of GTE, which is devoid of monomeric GTCs, including EGCG, resulted in reduced plasma levels of triglycerides and cholesterol, together with reduced gene expression of lipogenic enzymes, such as FASN, HMGR, and ACC, in mice, suggesting that green tea contains other components, in addition to EGCG, which may contribute to its anti-obesity effect [57]. This finding can be explained by the reduced expression of SREBPs caused by green tea constituents (Figure 2), since we also observed reduced gene expression of SREBP-1 and SREBP-2 [57].

SREBPs are regulated by AMPK, and it is known that green tea constituents upregulate AMPK by inducing the generation of ROS, as sufficient evidence has demonstrated that GTCs can act as pro-oxidants under certain conditions [7,8,58,59,60]. Metallic ions may also be associated with the pro-oxidant activity of GTCs (Figure 2) [61,62]. In addition, GTCs may increase ROS levels by decreasing the levels of antioxidant molecules and enzymes [63,64,65]. These pro-oxidant actions of GTCs can explain the findings related to their effect on gene and protein expression of enzymes and transcription factors involved in lipid metabolism (Figure 2).

In a rat model of galactosamine-induced liver injury, we found that catechin-rich green tea restored the galactosamine-induced increase in hepatic mRNA and plasma protein levels of TNF-α and IL-1β to normal levels [66]. These findings are compatible with those of other studies concerning the anti-obesity effects of green tea and GTCs. For example, Lu et al. carried out an experimental study in which rats were divided into two groups: control rats were fed a low-fat diet and obese rats were fed a high-fat diet. Their results showed that administration of GTCs to obese rats decreased the expression levels of 12 genes, including orexigenic genes, anorectic genes, such as IL-1β, and genes related to energy expenditure, than in the control rats [67].

A similar experiment showed that administration of 0.5% GTCs to obese rats increased the percentage of fat-free mass, bone mineral density, and bone strength, while it decreased the percentage of fat mass and serum levels of leptin, adiponectin, and proinflammatory cytokines, including TNF-α [68]. A more recent study also showed that EGCG supplementation attenuated the elevation in TNF-α protein in infiltrating CD68+ macrophages in the islets of rats fed a high-fat diet [69]. The results of a double-blind, placebo-controlled, human interventional trial showed that serum TNF-α and C-reactive protein levels were significantly lower, whereas total antioxidant status was higher, in the GTE group than those in the placebo group [70].

In these cases, the antioxidant action of GTCs is likely involved in their anti-obesity mechanism owing to the fact that ROS stimulate nuclear factor-κB, which in turn promotes the expression of proinflammatory cytokines, such as TNF-α and IL-1β [15,60]. GTCs may exert anti-obesity effects through suppression of TNF-α and IL-1β expression, since these inflammatory cytokines are known to upregulate the expression of SREBP-1 and stimulate the maturation of the SREBP-1 protein [71,72,73].

In contrast, as mentioned above, animal and cellular studies have shown that GTCs and other green tea constituents may stimulate the generation of ROS, which activate AMPK [7,60,74,75,76] and thus modulate the expression of genes and proteins involved in lipid metabolism. The pro-oxidant activity of green tea/GTE has also been shown in human studies. For example, Lambert et al. showed the generation of hydrogen peroxide in the oral cavity by either holding green tea in the mouth or chewing green tea leaves [77]. The pro-oxidant action of GTE has been suggested to be involved in hepatic toxicity associated with green tea supplements [78].

Therefore, it appears that two conflicting, i.e., antioxidative versus pro-oxidative mechanisms, are operating in the effect of green tea and GTCs. One possible explanation for these different actions is that the available concentration of green tea constituents can direct their action as either pro-oxidant or antioxidant [61,79,80]. In human lymphocytes, EGCG at a concentration range of 1–100 μM increased DNA strand breakage induced by bleomycin and hydrogen peroxide, but suppressed the breakage at a lower concentration range from 0.1 to 0.01 μM, suggesting that EGCG might have a dual function as a pro-oxidant and an antioxidant, depending on the concentration [79]. An experiment using non-obese type 2 diabetic Goto-Kakizaki rats showed that a diet containing 0.1% EGCG, but not 0.2% or 0.5% EGCG, reduced mRNA levels of TNF-α and IL-1β, and suppressed oxidative stress in the mesenteric adipose tissues, suggesting that the effects of EGCG are concentration-dependent [80]. The results of a chemiluminescence analysis showed that EGCG had a protective effect on calf thymus DNA at low concentrations (2–30 mM), while it enhanced oxidative DNA damage at higher concentrations (>60 mM) [61]. Further investigations are required to understand what is a determinant for GTCs to act as a pro-oxidant or an antioxidant.

## References

[1] Yang C.S., Chen G., Wu Q. (2014). Recent scientific studies of a traditional Chinese medicine, tea, on prevention of chronic diseases. J. Tradit. Complement. Med..

[2] Suzuki T., Miyoshi N., Hayakawa S., Imai S., Isemura M., Nakamura Y., Wilson T., Temple N.J. (2016). Health benefits of tea consumption. Beverage Impacts on Health and Nutrition.

[3] Hayat K., Iqbal H., Malik U., Bilal U., Mushtaq S. (2015). Tea and its consumption: Benefits and risks. Crit. Rev. Food Sci. Nutr..

[4] Kaibara E., Wilson W.S. (2008). Yojokun: Life Lessons from A Samurai.

[5] Wang S., Moustaid-Moussa N., Chen L., Mo H., Shastri A., Su R., Bapat P., Kwun I., Shen C.L. (2014). Novel insights of dietary polyphenols and obesity. J. Nutr. Biochem..

[6] Huang J., Wang Y., Xie Z., Zhou Y., Zhang Y., Wan X. (2014). The anti-obesity effects of green tea in human intervention and basic molecular studies. Eur. J. Clin. Nutr..

[7] Miyoshi N., Pervin M., Suzuki T., Unno K., Isemura M., Nakamura Y. (2015). Green tea catechins for well-being and therapy: Prospects and opportunities. Bot. Targets Ther..

[8] Yang C.S., Zhang J., Zhang L., Huang J., Wang Y. (2016). Mechanisms of body weight reduction and metabolic syndrome alleviation by tea. Mol. Nutr. Food Res..

[9] Sorice A., Guerriero E., Capone F., Colonna G., Castello G., Costantini S. (2014). Ascorbic acid: Its role in immune system and chronic inflammation diseases. Mini Rev. Med. Chem..

[10] Hughes L.A., Arts I.C., Ambergen T., Brants H.A., Dagnelie P.C., Goldbohm R.A., van den Brandt P.A., Weijenberg M.P., Netherlands Cohort Study (2008). Higher dietary flavone, flavonol, and catechin intakes are associated with less of an increase in BMI over time in women: A longitudinal analysis from The Netherlands Cohort Study. Am. J. Clin. Nutr..

[11] Vernarelli J.A., Lambert J.D. (2013). Tea consumption is inversely associated with weight status and other markers for metabolic syndrome in US adults. Eur. J. Nutr..

[12] Grosso G., Stepaniak U., Micek A., Topor-Mądry R., Pikhart H., Szafraniec K., Pająk A. (2015). Association of daily coffee and tea consumption and metabolic syndrome: Results from the Polish arm of the HAPIEE study. Eur. J. Nutr..

[13] Gyntelberg F., Hein H.O., Suadicani P. (2009). Sugar in coffee or tea and risk of obesity: A neglected issue. Int. J. Food Sci. Nutr..

[14] Takami H., Nakamoto M., Uemura H., Katsuura S., Yamaguchi M., Hiyoshi M., Sawachika F., Juta T., Arisawa K. (2013). Inverse correlation between coffee consumption and prevalence of metabolic syndrome: Baseline survey of the Japan Multi-Institutional Collaborative Cohort (J-MICC) Study in Tokushima, Japan. J. Epidemiol..

[15] Suzuki Y., Miyoshi N., Isemura M. (2012). Health-promoting effects of green tea. Proc. Jpn. Acad. Ser. B Phys. Biol. Sci..

[16] Rains T.M., Agarwal S., Maki K.C. (2011). Antiobesity effects of green tea catechins: A mechanistic review. J. Nutr. Biochem..

[17] Lin I.H., Ho M.L., Chen H.Y., Lee H.S., Huang C.C., Chu Y.H., Lin S.Y., Deng Y.R., He Y.H., Lien Y.H. (2012). Smoking, green tea consumption, genetic polymorphisms in the insulin-like growth factors and lung cancer risk. PLoS ONE.

[18] Nagao T., Komine Y., Soga S., Meguro S., Hase T., Tanaka Y., Tokimitsu I. (2005). Ingestion of a tea rich in catechins leads to a reduction in body fat and malondialdehyde-modified LDL in men. Am. J. Clin. Nutr..

[19] Matsuyama T., Tanaka Y., Kamimaki I., Nagao T., Tokimitsu I. (2008). Catechin safely improved higher levels of fatness, blood pressure, and cholesterol in children. Obesity (Silver Spring).

[20] Maki K.C., Reeves M.S., Farmer M., Yasunaga K., Matsuo N., Katsuragi Y., Komikado M., Tokimitsu I., Wilder D., Jones F. (2009). Green tea catechin consumption enhances exercise-induced abdominal fat loss in overweight and obese adults. J. Nutr..

[21] Cardoso G.A., Salgado J.M., Cesar Mde C., Donado-Pestana C.M. (2013). The effects of green tea consumption and resistance training on body composition and resting metabolic rate in overweight or obese women. J. Med. Food.

[22] Mähler A., Steiniger J., Bock M., Klug L., Parreidt N., Lorenz M., Zimmermann B.F., Krannich A., Paul F., Boschmann M. (2015). Metabolic response to epigallocatechin-3-gallate in relapsing-remitting multiple sclerosis: A randomized clinical trial. Am. J. Clin. Nutr..

[23] Boschmann M., Thielecke F. (2007). The effects of epigallocatechin-3-gallate on thermogenesis and fat oxidation in obese men: A pilot study. J. Am. Coll. Nutr..

[24] Wang H., Wen Y., Du Y., Yan X., Guo H., Rycroft J.A., Boon N., Kovacs E.M., Mela D.J. (2010). Effects of catechin enriched green tea on body composition. Obesity (Silver Spring).

[25] Basu A., Sanchez K., Leyva M.J., Wu M., Betts N.M., Aston C.E., Lyons T.J. (2010). Green tea supplementation affects body weight, lipids, and lipid peroxidation in obese subjects with metabolic syndrome. J. Am. Coll. Nutr..

[26] Shimoda H., Hitoe S., Nakamura S., Matsuda H. (2015). Purple tea and its extract suppress diet-induced fat accumulation in mice and human subjects by inhibiting fat absorption and enhancing hepatic carnitine palmitoyltransferase expression. Int. J. Biomed. Sci..

[27] Ashigai H., Taniguchi Y., Suzuki M., Ikeshima E., Kanaya T., Zembutsu K., Tomita S., Miyake M., Fukuhara I. (2016). Fecal lipid excretion after consumption of a black tea polyphenol containing beverage-randomized, placebo-controlled, double-blind, crossover study. Biol. Pharm. Bull..

[28] Chen I.J., Liu C.Y., Chiu J.P., Hsu C.H. (2016). Therapeutic effect of high-dose green tea extract on weight reduction: A randomized, double-blind, placebo-controlled clinical trial. Clin. Nutr..

[29] Nagao T., Meguro S., Hase T., Otsuka K., Komikado M., Tokimitsu I., Yamamoto T., Yamamoto K. (2009). A catechin-rich beverage improves obesity and blood glucose control in patients with type 2 diabetes. Obesity (Silver Spring).

[30] Li G., Zhang Y., Thabane L., Mbuagbaw L., Liu A., Levine M.A., Holbrook A. (2015). Effect of green tea supplementation on blood pressure among overweight and obese adults: A systematic review and meta-analysis. J. Hypertens..

[31] Li G., Zhang Y., Mbuagbaw L., Holbrook A., Levine M.A., Thabane L. (2014). Effect of green tea supplementation on blood pressure among overweight and obese adults: A protocol for a systematic review. BMJ Open.

[32] Bajerska J., Mildner-Szkudlarz S., Walkowiak J. (2015). Effects of rye bread enriched with green tea extract on weight maintenance and the characteristics of metabolic syndrome following weight loss: A pilot study. J. Med. Food.

[33] Brown A.L., Lane J., Holyoak C., Nicol B., Mayes A.E., Dadd T. (2011). Health effects of green tea catechins in overweight and obese men: A randomised controlled cross-over trial. Br. J. Nutr..

[34] Dulloo A.G., Duret C., Rohrer D., Girardier L., Mensi N., Fathi M., Chantre P., Vandermander J. (1999). Efficacy of a green tea extract rich in catechin polyphenols and caffeine in increasing 24-h energy expenditure and fat oxidation in humans. Am. J. Clin. Nutr..

[35] Hursel R., Viechtbauer W., Dulloo A.G., Tremblay A., Tappy L., Rumpler W., Westerterp-Plantenga M.S. (2011). The effects of catechin rich teas and caffeine on energy expenditure and fat oxidation: A meta-analysis. Obes. Rev..

[36] Mielgo-Ayuso J., Barrenechea L., Alcorta P., Larrarte E., Margareto J., Labayen I. (2014). Effects of dietary supplementation with epigallocatechin-3-gallate on weight loss, energy homeostasis, cardiometabolic risk factors and liver function in obese women: Randomised, double-blind, placebo-controlled clinical trial. Br. J. Nutr..

[37] Dostal A.M., Samavat H., Espejo L., Arikawa A.Y., Stendell-Hollis N.R., Kurzer M.S. (2016). Green tea extract and catechol-*O*-methyltransferase genotype modify fasting serum insulin and plasma adiponectin concentrations in a randomized controlled trial of overweight and obese postmenopausal women. J. Nutr..

[38] Dostal A.M., Arikawa A., Espejo L., Kurzer M.S. (2016). Long-term supplementation of green tea extract does not modify adiposity or bone mineral density in a randomized trial of overweight and obese postmenopausal women. J. Nutr..

[39] Moon H.S., Chung C.S., Lee H.G., Kim T.G., Choi Y.J., Cho C.S. (2007). Inhibitory effect of (−)-epigallocatechin-3-gallate on lipid accumulation of 3T3-L1 cells. Obesity (Silver Spring).

[40] Klaus S., Pültz S., Thöne-Reineke C., Wolfram S. (2005). Epigallocatechin gallate attenuates diet-induced obesity in mice by decreasing energy absorption and increasing fat oxidation. Int. J. Obes. (Lond.).

[41] Bose M., Lambert J.D., Ju J., Reuhl K.R., Shapses S.A., Yang C.S. (2008). The major green tea polyphenol, (−)-epigallocatechin-3-gallate, inhibits obesity, metabolic syndrome, and fatty liver disease in high-fat-fed mice. J. Nutr..

[42] Heber D., Zhang Y., Yang J., Ma J.E., Henning S.M., Li Z. (2014). Green tea, black tea, and oolong tea polyphenols reduce visceral fat and inflammation in mice fed high-fat, high-sucrose obesogenic diets. J. Nutr..

[43] Zheng G., Sayama K., Okubo T., Juneja L.R., Oguni I. (2004). Anti-obesity effects of three major components of green tea, catechins, caffeine and theanine, in mice. In Vivo.

[44] Unno T., Osada C., Motoo Y., Suzuki Y., Kobayashi M., Nozawa A. (2009). Dietary tea catechins increase fecal energy in rats. J. Nutr. Sci. Vitaminol..

[45] Grove K.A., Sae-tan S., Kennett M.J., Lambert J.D. (2012). (−)-Epigallocatechin-3-gallate inhibits pancreatic lipase and reduces body weight gain in high fat-fed obese mice. Obesity (Silver Spring).

[46] Yuda N., Tanaka M., Suzuki M., Asano Y., Ochi H., Iwatsuki K. (2012). Polyphenols extracted from black tea (*Camellia sinensis*) residue by hot-compressed water and their inhibitory effect on pancreatic lipase in vitro. J. Food Sci..

[47] Ikeda I., Tsuda K., Suzuki Y., Kobayashi M., Unno T., Tomoyori H., Goto H., Kawata Y., Imaizumi K., Nozawa A. (2005). Tea catechins with a galloyl moiety suppress postprandial hypertriacylglycerolemia by delaying lymphatic transport of dietary fat in rats. J. Nutr..

[48] Fei Q., Gao Y., Zhang X., Sun Y., Hu B., Zhou L., Jabbar S., Zeng X. (2014). Effects of Oolong tea polyphenols, EGCG, and EGCG3″Me on pancreatic α-amylase activity in vitro. J. Agric. Food Chem..

[49] Walkowiak J., Bajerska J., Kargulewicz A., Lisowska A., Siedlerski G., Szczapa T., Kobelska-Dubiel N., Grzymisławski M. (2013). Single dose of green tea extract decreases lipid digestion and absorption from a test meal in humans. Acta Biochim. Pol..

[50] Nishiumi S., Bessyo H., Kubo M., Aoki Y., Tanaka A., Yoshida K., Ashida H. (2010). Green and black tea suppress hyperglycemia and insulin resistance by retaining the expression of glucose transporter 4 in muscle of high-fat diet-fed C57BL/6J mice. J. Agric. Food Chem..

[51] Unno T., Sakuma M., Mitsuhashi S. (2014). Effect of dietary supplementation of (−)-epigallocatechin gallate on gut microbiota and biomarkers of colonic fermentation in rats. J. Nutr. Sci. Vitaminol..

[52] Murase T., Nagasawa A., Suzuki J., Hase T., Tokimitsu I. (2002). Beneficial effects of tea catechins on diet-induced obesity: Stimulation of lipid catabolism in the liver. Int. J. Obes. Relat. Metab. Disord..

[53] Ferré P., Foufelle F. (2007). SREBP-1c transcription factor and lipid homeostasis: Clinical perspective. Horm. Res..

[54] Murase T., Misawa K., Haramizu S., Hase T. (2009). Catechin-induced activation of the LKB1/AMP-activated protein kinase pathway. Biochem. Pharmacol..

[55] Santamarina A.B., Oliveira J.L., Silva F.P., Carnier J., Mennitti L.V., Santana A.A., de Souza G.H., Ribeiro E.B., Oller do Nascimento C.M., Lira F.S. (2015). Green tea extract rich in epigallocatechin-3-gallate prevents fatty liver by AMPK activation via LKB1 in mice fed a high-fat diet. PLoS ONE.

[56] Abe K., Okada N., Tanabe H., Fukutomi R., Yasui K., Isemura M., Kinae N. (2009). Effects of chronic ingestion of catechin-rich green tea on hepatic gene expression of gluconeogenic enzymes in rats. Biomed. Res..

[57] Yasui K., Paeng N., Miyoshi N., Suzuki T., Taguchi K., Ishigami Y., Fukutomi R., Imai S., Isemura M., Nakayama T. (2012). Effects of a catechin-free fraction derived from green tea on gene expression of enzymes related to lipid metabolism in the mouse liver. Biomed. Res..

[58] Suh K.S., Chon S., Oh S., Kim S.W., Kim J.W., Kim Y.S., Woo J.T. (2010). Prooxidative effects of green tea polyphenol (−)-epigallocatechin-3-gallate on the HIT-T15 pancreatic beta cell line. Cell. Biol. Toxicol..

[59] Li G.X., Chen Y.K., Hou Z., Xiao H., Jin H., Lu G., Lee M.J., Liu B., Guan F., Yang Z. (2010). Pro-oxidative activities and dose-response relationship of (−)-epigallocatechin-3-gallate in the inhibition of lung cancer cell growth: a comparative study in vivo and in vitro. Carcinogenesis.

[60] Hayakawa S., Saito K., Miyoshi N., Ohishi T., Oishi Y., Miyoshi M., Nakamura Y. (2016). Anti-cancer effects of green tea by either anti- or pro-oxidative mechanisms. Asian Pac. J. Cancer Prev..

[61] Tian B., Sun Z., Xu Z., Hua Y. (2007). Chemiluminescence analysis of the prooxidant and antioxidant effects of epigallocatechin-3-gallate. Asia Pac. J. Clin. Nutr..

[62] Farhan M., Khan H.Y., Oves M., Al-Harrasi A., Rehmani N., Arif H., Hadi S.M., Ahmad A. (2016). Cancer therapy by catechins involves redox cycling of copper ions and generation of reactive oxygen species. Toxins (Basel).

[63] Zhang H., Cao D., Cui W., Ji M., Qian X., Zhong L. (2010). Molecular bases of thioredoxin and thioredoxin reductase-mediated prooxidant actions of (−)-epigallocatechin-3-gallate. Free Radic. Biol. Med..

[64] Manohar M., Fatima I., Saxena R., Chandra V., Sankhwar P.L., Dwivedi A. (2013). (−)-Epigallocatechin-3-gallate induces apoptosis in human endometrial adenocarcinoma cells via ROS generation and p38 MAP kinase activation. J. Nutr. Biochem..

[65] Tao L., Forester S.C., Lambert J.D. (2014). The role of the mitochondrial oxidative stress in the cytotoxic effects of the green tea catechin, (−)-epigallocatechin-3-gallate, in oral cells. Mol. Nutr. Food Res..

[66] Abe K., Ijiri M., Suzuki T., Taguchi K., Koyama Y., Isemura M. (2005). Green tea with a high catechin content suppresses inflammatory cytokine expression in the galactosamine-injured rat liver. Biomed. Res..

[67] Lu C., Zhu W., Shen C.L., Gao W. (2012). Green tea polyphenols reduce body weight in rats by modulating obesity-related genes. PLoS ONE.

[68] Shen C.L., Cao J.J., Dagda R.Y., Chanjaplammootil S., Lu C., Chyu M.C., Gao W., Wang J.S., Yeh J.K. (2012). Green tea polyphenols benefits body composition and improves bone quality in long-term high-fat diet-induced obese rats. Nutr. Res..

[69] Cao Y., Bao S., Yang W., Zhang J., Li L., Shan Z., Teng W. (2014). Epigallocatechin gallate prevents inflammation by reducing macrophage infiltration and inhibiting tumor necrosis factor-α signaling in the pancreas of rats on a high-fat diet. Nutr. Res..

[70] Bogdanski P., Suliburska J., Szulinska M., Stepien M., Pupek-Musialik D., Jablecka A. (2012). Green tea extract reduces blood pressure, inflammatory biomarkers, and oxidative stress and improves parameters associated with insulin resistance in obese, hypertensive patients. Nutr. Res..

[71] Liu J. (2014). Ethanol and liver: Recent insights into the mechanisms of ethanol-induced fatty liver. World J. Gastroenterol..

[72] Zhang G., Li Q., Wang L., Chen Y., Wang L., Zhang W. (2011). Interleukin-1β enhances the intracellular accumulation of cholesterol by up-regulating the expression of low-density lipoprotein receptor and 3-hydroxy-3-methylglutaryl coenzyme A reductase in podocytes. Mol. Cell. Biochem..

[73] Lei X., Bone R.N., Ali T., Zhang S., Bohrer A., Tse H.M., Bidasee K.R., Ramanadham S. (2014). Evidence of contribution of iPLA2β-mediated events during islet β-cell apoptosis due to proinflammatory cytokine suggests a role for iPLA2β in T1D development. Endocrinology.

[74] Hwang J.T., Ha J., Park I.J., Lee S.K., Baik H.W., Kim Y.M., Park O.J. (2007). Apoptotic effect of EGCG in HT-29 colon cancer cells via AMPK signal pathway. Cancer Lett..

[75] Collins Q.F., Liu H.Y., Pi J., Liu Z., Quon M.J., Cao W. (2007). Epigallocatechin-3-gallate (EGCG), a green tea polyphenol, suppresses hepatic gluconeogenesis through 5′-AMP-activated protein kinase. J. Biol. Chem..

[76] Liu H.W., Chan Y.C., Wang M.F., Wei C.C., Chang S.J. (2015). Dietary (−)-Epigallocatechin-3-gallate supplementation counteracts aging-associated skeletal muscle insulin resistance and fatty liver in senescence-accelerated mouse. J. Agric. Food Chem..

[77] Lambert J.D., Kwon S.J., Hong J., Yang C.S. (2007). Salivary hydrogen peroxide produced by holding or chewing green tea in the oral cavity. Free Radic. Res..

[78] Yang C.S., Wang X., Lu G., Picinich S.C. (2009). Cancer prevention by tea: Animal studies, molecular mechanisms and human relevance. Nat. Rev. Cancer.

[79] Kanadzu M., Lu Y., Morimoto K. (2006). Dual function of (−)-epigallocatechin gallate (EGCG) in healthy human lymphocytes. Cancer Lett..

[80] Uchiyama Y., Suzuki T., Mochizuki K., Goda T. (2013). Dietary supplementation with (−)-epigallocatechin-3-gallate reduces inflammatory response in adipose tissue of non-obese type 2 diabetic Goto-Kakizaki (GK) rats. J. Agric. Food Chem..

